# A Case of Uncorrected Tetralogy of Fallot Undiagnosed Until Adulthood and Presenting With Polycythemia

**DOI:** 10.14740/cr374e

**Published:** 2014-12-04

**Authors:** Ercan Gunduz, Ahmet Gorgel, Recep Dursun, Hasan Mansur Durgun, Habip Cil, Mustafa Icer, Yilmaz Zengin

**Affiliations:** a Department of Emergency Medicine, Faculty of Medicine, Dicle University, Diyarbakir, Turkey; b Department of Endocrinology, Diyarbakir Education and Research Hospital, Diyarbakir, Turkey; c Department of Cardiology, Faculty of Medicine, Dicle University, Diyarbakir, Turkey

**Keywords:** Congenital heart disease, Polycythemia, Tetralogy of Fallot, Hemoglobins

## Abstract

Congenital heart defects with right-to-left shunt are one of the hypoxia-related causes of acquired secondary polycythemia (SP). Tetralogy of Fallot (ToF) is the most common congenital cyanotic heart disease in children. Cases of uncorrected ToF in adult ages are rare. This paper reports a woman detected with elevated hemoglobin level during routine tests performed for infertility therapy and subsequently diagnosed SP with related ToF.

## Introduction

Polycythemia may arise secondary to myeloproliferative disorders (e.g. polycythemia vera) or, more commonly, as a result of non-clonal increase in the number of red blood cells regulated by erythropoietin (secondary polycythemia (SP)). SP may be categorized into acquired or congenital types. The acquired SP includes the erythropoietin-associated (e.g. hypoxia, pathological erythropoietin production) or non-erythropoietin-associated (e.g. drugs, posttransplant erythrocytosis) forms. Congenital cardiac disorders with right-to-left shunt are one of the hypoxia-related causes of acquired SP [1]. Tetralogy of Fallot (ToF) is the most common congenital cyanotic heart disease in children. Cases of uncorrected ToF in adult ages are rare [2]. This paper reports a woman detected with elevated hemoglobin (Hgb) level during routine tests performed for infertility therapy and subsequently diagnosed with ToF.

## Case Report

**Figure 1:**
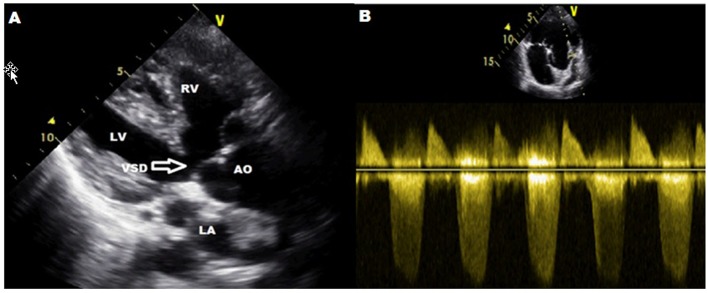
(A) Parasternal long axis view shows aortic dextroposition, ventricular septal defect, right ventricular hypertrophy. (B) Parasternal short axis view. Sample volume was placed on the right ventricular outflow tract and a 70 mm Hg gradient was calculated. LA: left atrium; LV: left ventricular; RV: right ventricular; AO: aortic valve; VSD: ventricular septal defect.

A 29-year-old woman was detected with elevated Hgb and hematocrit (Hct) levels during routine tests performed for infertility therapy, and underwent serial phlebotomies for polycythemia at an outside center 1 year ago. She admitted to our center on her own will and was hospitalized for advanced examination. She had no active complaints, nor had she any past or family history of any disease. On physical examination, her general status was well; she had a blood pressure of 115/85 mm Hg, pulse rate of 92 bpm, and respiratory rate of 24/min. She had perioral cyanosis and, on cardiac examination, 3/6 pansystolic murmur at the mesocardiac region. Blood tests performed at an outside center were as follows: Hgb: 21.5 g/dL, Hct: 64.4%, MCV: 92.2 f/L, MCH: 30.6 pg, MCHC: 31.4 g/dL, platelet: 214,000/μL, WBC: 6,600/μL, RBC: 7 × 10^6^/μL, urea: 20 mg/dL, creatinine: 0.79 mg/dL, AST: 21 U/L, ALT: 24 U/L, uric acid: 7.1 mg/dL, LDH: 265 U/L, vitamin B12: 170 pg/ml (155 - 982), ferritin: 126 ng/mL, serum erythropoietin (EPO): 38 mU/mL (10 - 30). An arterial blood gas analysis performed as an initial step to make the differential diagnosis of polycythemia revealed the following: pH: 7.46, pO_2_: 40.4 mm Hg, pCO_2_: 29.3 mm Hg, HCO_3_: 22.8 mEq/L, SO_2_: 80.4%. An electrocardiogram showed sinus rhythm with right axis deviation and right bundle branch block. Echocardiography revealed an ejection fraction of 60% with normal left ventricular systolic functions, a perimembranous ventricular septal defect (VSD), dextraposition of aorta, right ventricular dilatation and hypertrophy, and severe pulmonary infundibular stenosis (Fig. 1). Right and left heart catheterization and coronary angiography were performed. The cardiac catheterization excluded the Eisenmenger syndrome. Coronary angiography did not reveal any coronary artery anomalies. A joint cardiology and cardiovascular surgery committee decided a total corrective surgery to be carried out. In the control visit 6 months later the following laboratory data were obtained: Hgb: 12.5 g/dL, Hct: 37.2%, MCV: 86.7 f/L, MCH: 29.1 pg, MCHC: 30.3 g/dL, platelet: 149,000/μL, WBC: 4,400/μL, RBC: 4.29 × 10^6^/μL.

## Discussion

Polycythemia may arise secondary to myeloproliferative disorders (e.g. polycythemia vera) or, more commonly, as a result of non-clonal increase in the number of red blood cells regulated by erythropoietin (SP). SP has acquired and congenital subtypes [1]. The acquired forms include erythropoietin-associated and non-erythropoietin-associated forms. Congenital heart defects with right-to-left shunt are one of the hypoxia-related causes of acquired SP. Erythropoietin-mediated and hypoxia-dependent SP is characterized by an elevated erythropoietin level despite increased Hgb level. In SP not dependent on hypoxia, on the other hand, erythropoietin level is normalized after Hgb level has been elevated [3].

Our patient, a 29-year-old woman who was newly diagnosed with ToF, had a slightly increased erythropoietin level. Serum erythropoietin level cannot reliably distinguish SP causes from each other. In acquired SP, arterial hemoglobin oxygen saturation (SO_2_%) should be measured as the initial laboratory test. Renal and central nervous system imaging (in an attempt to detect an erythropoietin secreting tumor) should be considered when no central condition associated with hypoxia could be diagnosed. In our case the blood gas analysis was compatible with central hypoxia and thus the above-mentioned imaging tests were skipped.

In SP, the severity and the rate of thrombotic and non-thrombotic complications are substantially lower than those in polycythemia vera. Thus, in SP, prophylactic phlebotomy is recommended at a higher target Hct level (≥ 65) than the target level of polycythemia vera (40-45%) and in moderate-to-severe symptoms [3]. A study reported a patient with uncorrected ToF with SP at adulthood, said patient having underwent phlebotomy sessions at a regular basis despite an Hct level < 65% [4]. We did not consider phlebotomy in our patient as she had no signs or symptoms of hyperviscosity or any thrombotic or non-thrombotic complications, nor had she an Hct level > 65%.

Although ToF usually manifests with cyanosis in childhood, it appears with exercise intolerance and cyanosis in adults. As shown by previous reports, early total corrective surgery is the best treatment option. Majority of untreated patients probably die during childhood [5]. Pulmonary embolism, brain abscess, and thromboembolic events are the leading causes of death. In patients who underwent total corrective surgery, on the other hand, the long-term survival rate is excellent, with a 35-year survival rate of 85% [6].

Neurological manifestations may present with arterial and venous stroke, syncope, and seizure attacks. The expected clinical manifestation is usually the occurrence of cerebral venous thrombosis. While stroke has been linked to hyperviscosity and microstasis in children, it has been related to conventional risk factors in adults, such as phlebotomy, microcytosis, hypertension, diabetes mellitus, and rhythm disorders. Uncorrected ToF is only rarely encountered in adulthood. Only 1% of the untreated patients can survive by the age 50 years. In contrast, 74% of those who are operated can survive until that age [7]. The oldest uncorrected ToF in the literature aged 86 was reported from USA [8]. In our country, the oldest uncorrected ToF reported by Yokusoglu et al was 68 years old [9].

In conclusion, our patient was diagnosed with uncorrected ToF by a systemic examination during the differential diagnosis of polycythemia. This case highlights the importance of a systematic approach before proceeding with advanced investigations and treatment modalities in patients with polycythemia. A simple arterial blood gas analysis prevents unnecessary tests and treatments and may well diagnose an important pathology, i.e. uncorrected ToF which is rarely seen in adult population.
